# The Typhoid Carrier
*Dr. J. C. G. Ledingham's Report to the Local Government Board on the enteric fever "carrier"; being a review of current knowledge on this subject. (London : Wyman and Sons, Ltd. 1910. Price 1s.)


**Published:** 1910-12-10

**Authors:** 


### SPECIAL ARTICLE.

### THE TYPHOID CARRIER.*

Despite the comparatively recent discovery of the importance of the "carrier" (or person who, although apparently in good health, carries the infectious material of enteric fever in his system, whence it may be given off either in the urine or in the stools) as an agent in the dissemination of enteric fever, the literature of the subject has already attained large dimensions. It has been the object of Dr. J. C. Ledingham in his report to the Local Government Board to summarise the current knowledge of this subject, and the result is the present volume, which is in every way worthy of careful study by those interested in hygiene and public health.

### Its Discoverer.

The monograph is divided into fifteen chapters, the first of which is devoted to an introductory, from which we gather that to Robert Koch (1902) belongs the credit of insisting on the necessity of rendering the typhoid patient innocuous to those in contact with him by efficient disinfection of the excreta, both during the attack and subsequently, as well as by inquiry into the supervision of ambulant and abortive cases, and especially of those anomalous types met with so frequently in children. On this observer's recommendations, bacteriological stations were founded in typhoid-ridden districts, especially in South-west Germany, for the purpose of testing the validity of his dictum that the chief source of infection was to be found in man himself. The discovery of the typhoid carrier was the direct outcome of bacteriological research carried out in these institutes. It must not be forgotten, however, that Horton-Smith (1900), two years before Koch's address on the subject, had called attention to the ætiological importance of the chronic urinary carrier. According to Klinger (1909), there were in existence in 1907 eleven of these bacteriological stations with a staff of 35 bacteriologists attached to them, and with a population of 3,186,000 under their care. To ensure uniformity of action and co-operation among them, an "Imperial Commissioner for the typhoid campaign in the south-western portion of the Empire" was appointed in 1904, with headquarters at Saarbrücken. A District Commissioner was allotted to Bavaria. The respective municipal authorities lent their aid to these officials in the execution of their duties relative to the general hygienic conditions of the various districts. To the stations was allotted the task of examining bacteriologically suspected material from patients with a view to accurate diagnosis. Power was given to the scientists to prosecute inquiries on the spot with the local authorities, while access was granted them to police records, dispensary lists, the official death-notification statistics, etc.; and they were also allowed to obtain information from the clergy, the school teachers, and the midwives. Every three months a report had to be made to the district chairman and the Imperial Commissioner, who presented a return to the Imperial Board of Health.

### Notification Rules.

With regard to notification, diagnosis, and prevention of spread of the disease, the following are some of the instructions laid down: 1. Notification must be made not only of undoubted cases of typhoid fever, but also of suspected cases. 2. In the diagnosis of the disease the assistance of the director of the bacteriological station as well as of the official

* Dr. J. C. G. Ledingham's Report to the Local Government Board on the enteric fever "carrier"; being a review of current knowledge on this subject. (London: Wyman and Sons, Ltd. 1910. Price 1s.)

health officers should be requisitioned. These should have access to the sick-room as far as the medical attendant considers advisable in the interest of his patient. 3. Among the measures to prevent the spread of the disease, the removal of the sick and of suspected cases is provided for. Persons excreting typhoid bacilli, but who present no symptoms, or only slight ones, of typhoid fever, and convalescents who are still discharging the specific bacilli, should as far as possible be dealt with as sick persons. Where no legal right exists for this purpose oral and written instructions should be given enjoining the necessity for the disinfection of excreta and for personal cleanliness so as to prevent reinfection and the spread of the disease to others. Typhoid patients should be taken to hospital and retained there until two examinations, at weekly intervals, have demonstrated the absence of bacilli in their excreta, and wherever possible the number of such examinations should be extended to three.

### Facts about the Carriers.

In his second chapter Dr. Ledingham discusses the general facts regarding carriers, such as age, sex, frequency, etc. He shows that the early work of the institutes abundantly verified Koch's contentions, and led Frosch to state a hypothesis that the *B. typhosus* could lead a prolonged saprophytic existence in the human intestine, a hypothesis which was definitely established by Drigalski in 1903-4. In a case which is the first recorded of a chronic carrier traced from convalescence, this last observer found at the end of nine months that the bacilli were present and constituted 50 to 60 per cent. of the total intestinal flora. The mass of facts which has accumulated since then shows that in proportion to the efficiency of the bacteriological methods employed so do the instances become more numerous in which the bacillus can be demonstrated in the fæces at all stages of the disease. As regards the urine, most observers agree that the bacillus can be detected in about 25 per cent. of cases, but usually only in the later stages of the disease after defervescence or in late convalescence. It has however, been found at the period of the eruption of the rose-spots. The author next proceeds to investigate figures given by Lentz (1905) with reference to the prevalence of carriers among convalescent typhoid patients at the Institute in Idar. Out of 400, six continued to excrete bacilli for a longer period than ten weeks from the beginning of the disease or of a relapse, but none of these remained infective after thirteen months. Twelve others did not rid themselves of bacilli, but became chronic carriers. His percentages thus worked out at 1.5 for temporary and 3 for chronic carriers. Extending his inquiries among the patients' relatives he found twenty-two other carriers, of whom nineteen were females, in three of which last gall-stone disease subsequently developed.

### The Classification of Carriers.

Dr. Ledingham considers Sacquepée's classification of carriers the best. In this the patients are divided into three groups. The first comprises "precocious carriers"—*i.e.* those who have taken the typhoid bacillus into the system but as yet present no symptoms. This group consists of carriers in the incubation stage. The second group contains two sub-groups: (*a*) "Convalescent carriers," in whom the excretion ceases before the end of the third month; and (*b*) "Chronic carriers," in whom the excretion lasts for an indefinite period. The third group consists of "healthy or paradoxical carriers," those who have never had symptoms of typhoid fever but may excrete the bacillus over an indefinite period. The distinction between the first and third groups may at times be very difficult. Klinger (1906) examined 1,700 persons and found fifteen carriers, of whom seven were females. The fifteen varied in age from eighteen months to sixty years. Eleven had no clinical symptoms, either before or after examination. Only four were found to be chronic carriers, two of each sex. Out of a further 482 cases examined during convalescence 11.4 per cent. excreted bacilli, but only eight, or 1.7 per cent., became chronic carriers. In a later communication he reports that of 604 convalescents examined he found eighty temporary carriers (seventy, or 11.6 per cent., intestinal, and ten, or 1.7 per cent., urinary), while six, or 1 per cent., became chronic carriers. He states that urotropin cured all the temporary carriers. Bruckner (1910) found twelve carriers out of 316 persons examined, who had had typhoid fever some years previously. This shows, in his opinion, the necessity of late control examinations. Semple and Greig's report on work carried out at the Kasauli Research Institute in India is next examined. These observers found ten patients out of eighty-six, or 11.6 per cent., excreting bacilli in the urine or fæces for periods longer than six weeks after defervescence. Aldridge (1909) found six out of 190 men, or 3.1 per cent., excreting the bacilli more than six months after defervescence. The author goes on to refer to other outbreaks in France, Japan, America, and Germany, which all bear out the contention that a considerable proportion of convalescents become chronic carriers, and that by far the largest number of the latter are of the female sex. Children appear to form a large proportion of transitory but only a small proportion of chronic carriers. It would seem to be demonstrated that a preponderant number of contact infections take place during the early period of the typhoid infection, which is the same as saying that the typhoid patient is most dangerous during the incubation period.

### The Danger of Carriers.

The author devotes his next four chapters to instances of the infectivity of carriers. It is impossible in the space at our disposal to follow him through his interesting account; but he abundantly proves the influence of carriers in maintaining the endemicity of typhoid fever in individual houses and streets or groups of houses in isolated country districts. He shows how infection may be carried by water polluted from imperfect water-closet arrangements, by food-stuffs handled by carriers before consumption by others, and by contact with imperfectly disinfected excreta of carriers, and gives examples collected from the literature of each mode of propagation of the disease. He gives a full account of milk-borne cases, cases in institutions and in the Army. Chapter VII. is devoted to the pathogenesis of the typhoid-carrying state, with particular reference to the intestinal carrier. In this chapter, after stating that it was long known that typhoid bacilli could be found in cases of cholecystitis, cholelithiasis, bone abscesses, etc., the author discusses the incidence of gall-stone disease among carriers. He quotes Forster's (1908) figures, who found that out of 194 convalescent carriers who continued to carry the bacilli after convalescence, 29 per cent. were men, 45 per cent. women, and 26 per cent. children. On the other hand, out of 173 chronic carriers who excreted bacilli from one to 30 years after the primary attack, 79 per cent. were women, 17 per cent. men, and 4 per cent. children. This striking preponderance of female over male chronic carriers suggests an analogy to the relative gall-stone incidence in the female and male. As a matter of fact, 25, or 14 per cent., of the 173 carriers presented gall-stone symptoms during life, the remaining 86 per cent. betraying no such symptoms. This, again, is in accordance with the dictum that in most cases gall-stones present no symptoms during life. The question as to whether gall-stones in some cases result from typhoid fever, or whether the bacilli attach themselves to preformed stones, is not yet settled, but the author sets forth the chief arguments advanced on both sides. In the eighth chapter the physical disabilities of the carrier state are discussed, and it would seem that, apart from gall-bladder trouble, the typhoid carrier is little inconvenienced by his condition. Occasionally he complains of headaches, especially if he is a urinary carrier, and in some cases periodic intestinal disturbances have been observed in carriers of the intermittent type. As a rule, however, his bodily health is good.

### Notes on Treatment.

The treatment of intestinal carriers, which is gone into in the next chapter, is unfortunately attended by very unsatisfactory results. Intestinal antiseptics exert but a momentary effect on the number of bacilli excreted. Salol, beta-naphthol, calomel, bile and its salts, turpentine oil, sodium bicarbonate in large doses, chloroform, etc., have all been tried. At best it can be said at present that if pushed to their physiological limits they offer some slight prospect of success. Lactic acid milk has been tried, but is in the main of little use, though in one or two cases a cure has been reported after its use. Operative measures, such as removal or drainage of the gall-bladder, have not been carried out in a sufficiently large number of cases to warrant an opinion being formed as to their efficacy. A few cases are on record, however, in which such proceedings appear to have done good, while in others no such result was obtained. Urinary carriers, which are next dealt with, are fortunately not so frequently met with as intestinal ones, for the danger of spreading infection by the urine is greater than by the fæces. The question of their treatment has not, however, been settled in a more satisfactory manner than in the case of intestinal carriers. Urotropin has not yielded satisfactory results. Hetralin is of little use. Borovertin has proved useful in one recorded case of a cure by its exhibition. The patient had received first a utropin and then a hetralin course of treatment, without much success. The borovertin was then tried, and after doses of six grams daily in one-gram doses for a period of over a month the urine was found free from bacilli after numerous examinations. This drug is therefore worthy of a trial, either alone or after a preliminary course of urotropin. Vaccines of a homologous strain have proved successful in one case, but only after rendering the urine alkaline by sodium lactate administered by the mouth. In the next chapter an exhaustive account is given of the diagnostic methods employed in the search for carriers. This includes a description of the various media employed by different observers for the isolation of the bacillus, and questions of space forbid our entering into the matter here. The rest of the monograph deals with the discussion of the general question of carrier-infectivity, with statistical data regarding the infectivity of carriers, the immunity question in carriers, and the various measures either employed or suggested to diminish the spread of infection by carriers. In the absence of some efficient therapeutic method all attempts to cope with the spread of infection are worthy of serious consideration. Mayer (1909) suggests the foundation of convalescent homes for recovered typhoid cases, where, by suitable treatment, dietetic and otherwise, efforts would be made to encourage the physiological excretion of bacilli until possibly complete cessation results. There can be no doubt that such institutions would prove useful in many ways, for those in a potentially infective condition could be thoroughly instructed as to the precautions they should take on return to civil life. In addition, they would furnish opportunities for the collection of full and accurate statistics regarding the duration and severity of the disease, the diet employed in convalescence and in the home, the muscular power of the patient, and the bacteriological results. The monograph closes with a number of sets of instructions issued by various authorities to patients when discharged from hospital, and to chronic carriers. An appendix contains a full bibliography of the authorities upon which the author's findings are based.

